# A Cross-Sectional Analysis of the Development of Response Inhibition in Children with Chromosome 22q11.2 Deletion Syndrome

**DOI:** 10.3389/fpsyt.2013.00081

**Published:** 2013-08-07

**Authors:** Heather M. Shapiro, Ling M. Wong, Tony J. Simon

**Affiliations:** ^1^Department of Psychiatry and Behavioral Sciences, MIND Institute, University of California Davis, Sacramento, CA, USA

**Keywords:** 22q11.2 deletion syndrome, response inhibition, executive function, childhood cognitive development, developmental disorders

## Abstract

Chromosome 22q11.2 deletion syndrome (22q11.2DS) is a neurogenetic disorder that is associated with cognitive impairments and significantly elevated risk for developing schizophrenia. While impairments in response inhibition are central to executive dysfunction in schizophrenia, the nature and development of such impairments in children with 22q11.2DS, a group at high risk for the disorder, are not clear. Here we used a classic Go/No-Go paradigm to quantify proactive (anticipatory stopping) and reactive (actual stopping) response inhibition in 47 children with 22q11.2DS and 36 typically developing (TD) children, all ages 7–14. A cross-sectional design was used to examine age-related associations with response inhibition. When compared with TD individuals, children with 22q11.2DS demonstrated typical proactive response inhibition at all ages. By contrast, reactive response inhibition was impaired in children with 22q11.2DS relative to TD children. While older age predicted better reactive response inhibition in TD children, there was no age-related association with reactive response inhibition in children with 22q11.2DS. Closer examination of individual performance data revealed a wide range of performance abilities in older children with 22q11.2DS; some typical and others highly impaired. The results of this cross-sectional analysis suggest an impaired developmental trajectory of reactive response inhibition in some children with 22q11.2DS that might be related to atypical development of neuroanatomical systems underlying this cognitive process. As part of a larger study, this investigation might help identify risk factors for conversion to schizophrenia and lead to early diagnosis and preventive intervention.

## Introduction

Adaptive behavior in a dynamic world relies heavily on cognitive control, defined as the ability to suppress irrelevant thoughts and actions while strengthening others (1). An integral factor in successful cognitive control is response inhibition, which supports the suppression of a prepotent response that might be irrelevant or inappropriate in a given context. Evidence suggests that response inhibition improves throughout child development and into adulthood (2–4), likely due to the development of supporting neural architecture (5, 6). Additionally, response inhibition is often impaired in individuals with developmental disabilities or psychopathology (7). Individuals with schizophrenia perform more poorly on response inhibition tasks when compared to healthy controls (8, 9), and it is believed that aberrant response inhibition might contribute to some of the general neuropsychological impairments seen in the disorder. Evidence also suggests that impairments in control processes might be present in individuals long before conversion to schizophrenia (10–12), as well as in their unaffected first-degree relatives (13). Thus, impairments in inhibitory control are considered a key component of the intermediate phenotype of the disorder (14, 15).

Individuals with chromosome 22q11.2 deletion syndrome (22q11.2DS) represent a population with a genetically conferred risk for developing schizophrenia that is significantly increased relative to the general population. Approximately 30% of children with 22q11.2DS will develop schizophrenia by adulthood (16), rendering it the highest genetic risk factor for the disorder after having two parents or a monozygotic twin with schizophrenia. Additionally, elevated rates of psychotic symptoms have been detected in children with 22q11.2DS (17, 18).

Chromosome 22q11.2DS results from a 1.5- to 3-megabase microdeletion on the long (q) arm of chromosome 22 (19, 20) and occurs in approximately 1 in 2000–4000 live births (21, 22). Children with this disorder have mild to moderate intellectual impairments (median full scale IQ 70 ± 15) (23) and a cognitive profile with difficulties on a range of functions including attention and numerical processing (24), as well as cognitive control (25, 26). With respect to inhibitory control, impairments have been demonstrated in children with 22q11.2DS on tasks requiring interference control (25) and oculomotor inhibition (27).

Response inhibition, a component of inhibitory control defined as the ability to suppress a prepotent motor response, has not been well characterized in children with 22q11.2DS. One study examined response inhibition in adolescents with 22q11.2DS using a Go/No-Go task during functional magnetic resonance imaging (fMRI) (28). While no behavioral group differences were found, individuals with 22q11.2DS demonstrated significantly greater brain activation in the left superior and inferior parietal lobes, suggesting compensatory mechanisms might underlie typical behavioral performance. For the blocked design of this fMRI study, the Go/No-Go task consisted of two conditions: a block of all Go trials, on which participants responded on every trial (control condition), and a block of trials with 50% Go/No-Go probability (experimental condition). It is possible that this relatively coarse design, necessary for power in a fMRI study, might obscure more subtle, specific behavioral impairments in response inhibition in 22q11.2DS.

In the present study, our goal was to examine response inhibition in children with 22q11.2DS by using a Go/No-Go task that was designed to parametrically manipulate task difficulty, as well as test specific component processes of response inhibition. Participants were required to press a button in response to a frequently occurring target (“Go” trial), and to avoid pressing the button in response to an infrequent target (“No-Go” trial). Task difficulty was manipulated by varying the number of Go trials leading up to a No-Go trial, which has previously been shown to affect response prepotency and thus ability to withhold a response (6). Additionally, this Go/No-Go task supported the assessment of multiple component processes of response inhibition. First there is *proactive* response inhibition, which involves appropriate monitoring of task context and preparation leading up to a successful inhibitory response. This can be measured by response time on Go trials and can manifest as a relative slowing of sequential Go trials leading up to a No-Go trial (29). Secondly, there is *reactive* response inhibition, which is the actual inhibitory response once instructed by a stop signal, and is measured by accuracy on No-Go trials (30). Finally, the Go/No-Go task is sensitive to conflict monitoring, which modulates behavior in response to perceived performance and is typically demonstrated by a relative slowing on the first Go trial following an incorrect relative to a correct No-Go trial (31).

Understanding component processes of response inhibition in children with 22q11.2DS is important not only for better characterizing cognitive function in this population, but also for examining a cognitive process that is considered to be a component of the schizophrenia intermediate phenotype (13, 14). Given the genetic predisposition for a significantly elevated schizophrenia risk in 22q11.2DS, individual performance patterns of response inhibition might help describe individuals at relatively greater or lesser risk for conversion. Since we did not collect measures of psychosis on the current sample of participants, we were unable to directly test this hypothesis. However, examining performance variability of a process that is considered to be part of an intermediate phenotype for schizophrenia is an important first step. Additionally, age-related patterns of response inhibition development might highlight individuals with more or less typical response inhibition. Thus, in addition to characterizing the nature of response inhibition in 22q11.2DS, another goal of the current study was to examine the development of response inhibition through mid to late childhood in 22q11.2DS by conducting a cross-sectional analysis in school-aged children (7–14 years) with 22q11.2DS and age-matched typically developing (TD) children.

Based on previous findings of executive dysfunction and cognitive disinhibition, we hypothesized that children with 22q11.2DS would demonstrate impaired performance on the Go/No-Go task when compared to age-matched TD children, and that there would be age-related group differences. Specifically, we predicted that (1) proactive and reactive response inhibition might be differentially sensitive to group differences, and (2) response inhibition would improve with age in TD children but less so in children with 22q11.2DS.

## Materials and Methods

### Participants

Forty-seven children with 22q11.2DS [mean age = 11.5(2.5) years; 17 female and 30 male] and 36 TD comparison children [mean age = 10.7(2.3) years; 21 female and 15 male], from 7 to 14 years of age, participated in the study. Participants were recruited to participate in the study by advertisement, and by the UC Davis Subject Tracking System, a secure, voluntary database that houses the names of families interested in participating in research studies. Documentation of the deletion was provided by parents/guardians during the pre-screening process. Data on IQ from the Wechsler Intelligence Scale for Children – 4th edition (WISC-IV) (32) or the Wechsler Abbreviated Scale of Intelligence (WASI) (33) was available from a subset of participants: 36 children with 22q11.2DS and 22 TD participants. Full-scale IQ (FSIQ) ranged from 54 to 103 for children with 22q11.2DS [mean = 75.1(12.0)] and 80–137 for TD children [mean = 110.0(12.0)]. Complete demographic information and group gender composition can be found in Table 1. A subsample of the study participants (10 with 22q11.2DS and 8 TD) performed the cognitive task at a conference where they did not complete the WISC-IV or the WASI, thus contributing to incomplete IQ data. At the conference, participants were recruited by advertisements and direct person-to-person solicitation. Participants were excluded if they performed at lower than 75% accuracy when responding to the frequently occurring Go stimuli in the behavioral paradigm. Four participants with 22q11.2DS and one TD participant were excluded on this basis, resulting in the final sample of 47 children with 22q11.2DS and 36 TD children that are described here. Additional exclusion criteria for both groups included head injury or other focal neurological abnormality. Exclusion criteria for TD participants were presence of learning or behavioral/psychiatric disorder. The parents of all participants provided written informed consent based on protocols approved by the Institutional Review Board at the University of California, Davis.

**Table 1:** **Demographic data on children with 22q11.2DS and TD children**.

		22q11.2DS				TD			
		Age in years		FSIQ		Age in years		FSIQ	
		*N*	Mean (SD)	*N*	Mean (SD)	*N*	Mean (SD)	*N*	Mean (SD)
Gender	Female	17	12.0 (2.3)	13	70.6 (10.5)	21	10.9 (2.5)	12	112.2 (14.9)
	Male	30	11.3 (2.7)	23	77.6 (12.3)	15	10.4 (2.1)	10	109.5 (7.6)
Total		47	11.5 (2.5)	36	75.1 (12.0)	36	10.7 (2.3)	22	111.0 (12.0)

### Task procedure

**Figure 1:**
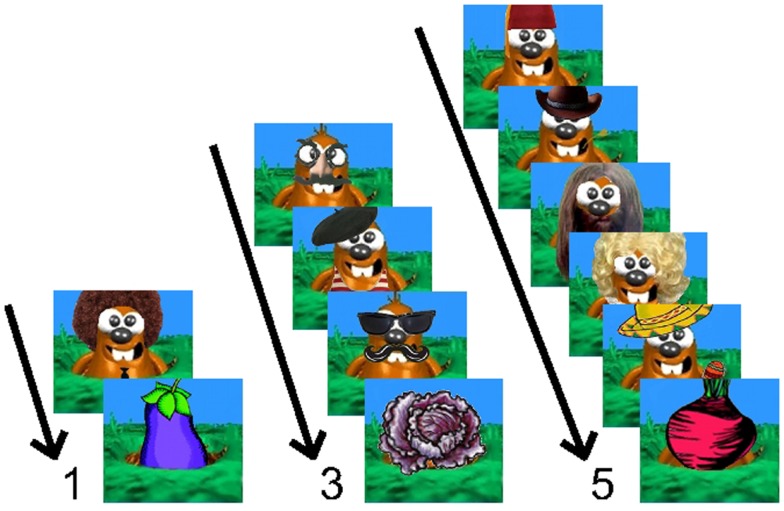
**Example trials from the “whack-a-mole” Go/No-Go task**. Children were instructed to press a button as quickly as possible when a cartoon mole appeared (Go trial), but to avoid pressing the button when a vegetable appeared (No-Go trial). Go trials appeared 75% of the time, while No-Go trials appeared the other 25% and were preceded by one, three, or five Go trials.

All participants completed a “whack-a-mole” version of a Go/No-Go response inhibition task (Figure 1). This experiment was adapted from Casey et al. (34) and stimuli were courtesy of Sarah Getz and the Sackler Institute for Developmental Psychobiology. Children viewed a computer screen from 60 cm and were told to press a button as quickly as possible when a cartoon mole appeared (Go trial). They were instructed to *not* press the button when a vegetable appeared (No-Go trial). During the demonstration period, participants were instructed to perform the task as quickly as they could while still responding as accurately as possible. Equal emphasis was placed on speed and accuracy. Go trials appeared 75% of the time, while No-Go trials appeared the other 25% and were preceded by one, three, or five Go trials. The duration of the stimuli was 1000 ms, with interstimulus intervals of 200, 500, or 750 ms. Participants completed 20 trials of each No-Go trial type (preceded by one, three, or five Go trials, respectively), randomized and distributed equally into four blocks. The experiment also contained 12 foil trials (No-Go trials preceded by two or four Go trials) in order to prevent learning of the pattern.

### Data analysis

Data were processed using MATLAB (version 7.8) to generate outcome variables from raw data. Mixed model regression analyses were used to determine the effects of between-subject variables (diagnosis group) and task variables (Go or No-Go trial type) on primary outcome measures (accuracy and response time). Gender was included as a predictor in all regression models. To examine the development of response inhibition, we included age as a regressor in additional models to examine age-related effects in a cross-sectional analysis.

For each outcome measure, we first conducted regressions on data from the two groups combined in order to examine between-group differences. Subsequently, we identified distinct trial types: five trial types for Go trials (one through five based on sequential order following a No-Go trial) and three trial types for No-Go trials (No-Go trials appearing after one, three, or five Go trials, respectively). We tested for Group × Trial Type interactions, and if any were identified, we then ran the regression models within each group separately in order to test for group-specific effects of trial type on performance. Finally, we corrected for multiple comparisons using false discovery rate (FDR). In the results below, we documented all of the results, both before and after the correction for multiple comparisons, in order to provide a most complete and comprehensive picture of response inhibition in 22q11.2DS.

## Results

### Proactive response inhibition did not differ between groups

**Figure 2:**
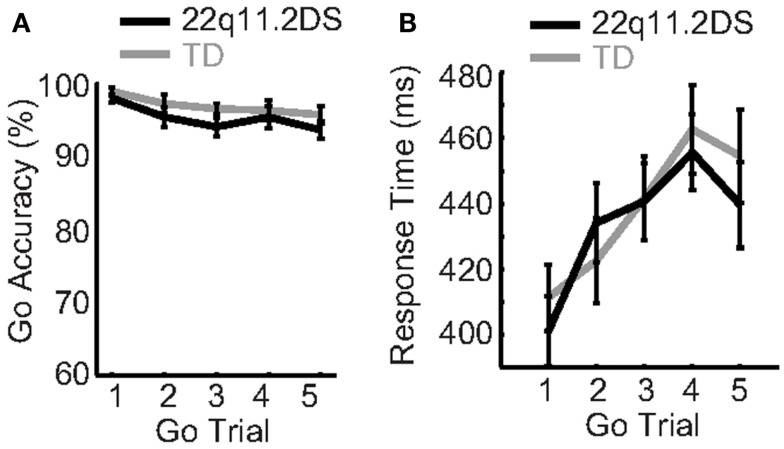
**Proactive response inhibition was typical in children with 22q11.2DS**. **(A)** Accuracy and **(B)** response time on Go trials did not differ between groups.

Proactive response inhibition, defined as the preparation prior to an upcoming inhibitory response, was measured by accuracy and response time (RT) on consecutive Go trials leading up to a No-Go trial. Diagnostic group, Go trial type (one through five based on sequential order following a No-Go trial), and gender were regressed on accuracy and RT. There was no Group × Trial Type interaction on accuracy [*F*(4, 234) = 0.25, *p* = 0.91; Figure 2A], but there was a Group × Trial Type interaction on RT [*F*(4, 234) = 3.24, *p* = 0.02; Figure 2B]. In order to further unpack the Group × Trial Type interaction on RT, we examined the effects of Go trial type on RT within each group separately. Both groups demonstrated a similar performance pattern, consisting of a relative slowing from the first up to the fourth Go trial following a No-Go trial [*F*(4, 140) = 25.6, *p* < 0.0001 for TD; *F*(4, 184) = 28.5, *p* < 0.0001 for 22q11.2DS; Figure 2B]. Additionally, the Group × Trial Type interaction on RT did not survive correction for multiple comparisons. Thus, we concluded that this interaction may be a spurious result due to noise that generates subtle group effects at different trial types, such as the slower RT in one group on the second Go trial, relative to a faster RT on the fifth Go trial. While accuracy was the same between groups and was not affected by trial type, both groups demonstrated a comparable and significant slowing of RT on consecutive Go trials, indicative of similar proactive inhibition between groups.

### Reactive response inhibition was aberrant in 22q11.2DS

**Figure 3:**
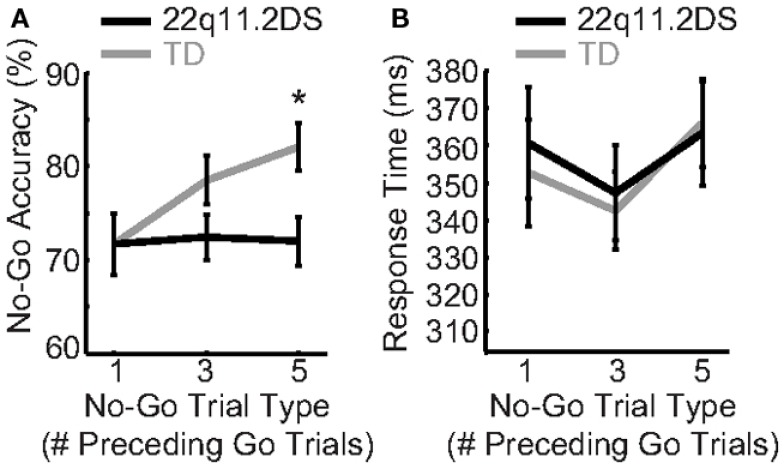
**Reactive response inhibition was atypical in children with 22q11.2DS**. **(A)** TD children demonstrated better No-Go accuracy as a function of more preceding Go trials, while children with 22q11.2DS did not demonstrate this pattern. **(B)** There were no group differences in response time on incorrect No-Go trials (false alarms).

Reactive response inhibition, defined as the implementation of an inhibitory response once instructed by a stop signal, was measured by accuracy on No-Go trials that were parametrically manipulated for difficulty (No-Go trials following one, three, or five Go trials, respectively). Diagnostic group, No-Go trial type, and gender were regressed on accuracy and RT. We found a significant Group × Trial Type interaction [*F*(2, 162) = 3.83, *p* = 0.02; Figure 3A]. In order to better understand this interaction, we next examined the effects of No-Go trial type within each group separately by regressing No-Go trial type on No-Go accuracy for each group. There was a significant effect of No-Go trial type on accuracy in TD children, such that when No-Go trials were preceded by increasing numbers of Go trials, TD children had greater accuracy [*F*(2, 70) = 7.07, *p* = 0.002; mean accuracy = 71.7(19.2), 78.5(15.5), and 82.1(15.5)% for one, three, and five preceding Go trials, respectively]. By contrast, children with 22q11.2DS demonstrated no change in performance across trial types [*F*(2, 92) = 0.05, *p* = 0.95; mean accuracy = 71.7(16.4), 72.4(16.5), and 72.0(18.0)% for one, three, and five preceding Go trials, respectively; Figure 3A]. The Group × Trial Type interaction on No-Go accuracy did not remain significant after accounting for multiple comparisons; however, within the TD group, the effect of Trial Type on No-Go accuracy survived this correction. Thus, it appears that the two groups have differential patterns of performance as a function of No-Go trial type.

In order to examine possible group differences in the error mechanism, we next examined RT on the incorrect No-Go trials, or false alarms. There were no effects of group, or a Group × Trial Type interaction (Figure 3B). Thus, the false alarm RT was the same between and within groups across No-Go trial types.

### Reactive response inhibition was impaired in older children with 22q11.2DS

**Figure 4:**
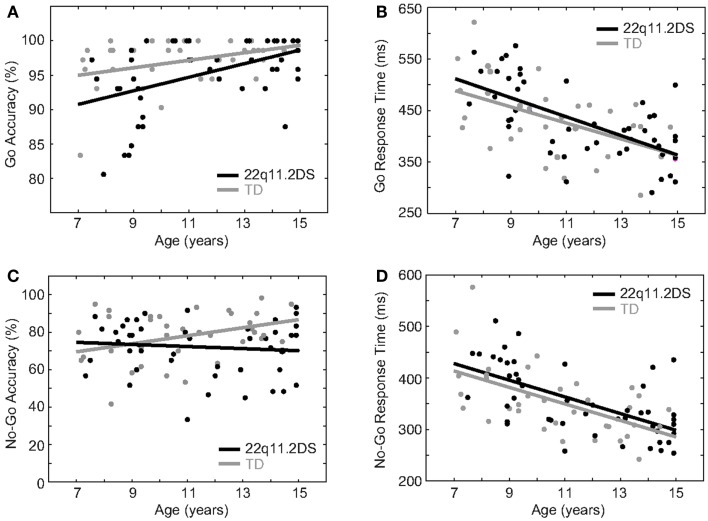
**Age effects on response inhibition**. **(A)** Improved accuracy and **(B)** faster RT were associated with age on Go trials. There were no group differences in these relationships. **(C)** Reactive response inhibition was better in older TD children relative to younger, but this was not the case in children with 22q11.2DS. **(D)** Both groups demonstrated a similar relationship of faster No-Go (false alarm) RT and age.

To examine the development of response inhibition in the two groups, we conducted a cross-sectional analysis in children with 22q11.2DS relative to TD children, all aged 7–14 years with no age difference between groups [*t*(77) = 1.03, *p* = 0.31]. To assess proactive inhibition, the following were regressed on Go accuracy and RT: diagnostic group, age, gender, and Go trial type. There were main effects of age, such that age predicted higher accuracy [*F*(1, 78) = 13.90, *p* = 0.0004] and faster RT [*F*(1, 78) = 39.20, *p* < 0.0001], although the Age × Group interaction was not significant (*p* = 0.26 and *p* = 0.77 for accuracy and RT, respectively; Table 2; Figures 4A,B). The age effects on accuracy and RT survived correction for multiple comparisons. Thus, accuracy was better and RT was faster in older individuals across both groups, and this pattern did not differ between groups.

**Table 2:** **Age effects on Go/No-Go performance**.

Outcome measure	Group × Age interaction	
	*F*	*p*
Percent accuracy on Go trials	1.15	0.29
RT on Go trials (ms)	0.23	0.63
Percent accuracy on No-Go trials	4.4	0.04*
RT on incorrect No-Go trials (ms)	0.00	1
Post-error processing difference (ms)	0.69	0.41

*Above are the test statistics from regressions of age against each outcome measure with both groups in the same model to examine group by age interactions that might represent group differences in developmental trajectories. Gender was included in all models*.

To examine age effects on reactive inhibition, the following were regressed on No-Go accuracy: diagnostic group, age, gender, and No-Go trial type. Collectively, there was no overall effect of age [*F*(1, 78) = 0.59, *p* = 0.45], but there was a significant Age × Group interaction on No-Go accuracy [*F*(1, 78) = 4.39, *p* = 0.04; Table 2; Figure 4C]. Within groups, there was a significant effect of age on accuracy in TD children, such that older TD children had higher No-Go accuracy [*F*(1, 33) = 4.9, *p* = 0.03]. By contrast, performance in children with 22q11.2DS did not differ with age [*F*(1, 44) = 0.45, *p* = 0.51]. Though the Age × Group interaction on No-Go accuracy did not remain significant after accounting for multiple comparisons, the main effect within the TD group survived this correction. Thus, while the TD children demonstrated an association between improved reactive inhibition and age, the children with 22q11.2DS did not. This appears due to a subgroup of the oldest children with 22q11.2DS that have lower levels of accuracy relative to others their age in either group.

We next examined age-related effects on No-Go RT. While there was a main effect of age, such that age predicted faster RT [*F*(1, 78) = 36.00, *p* < 0.0001], there were no interactions of Age × Group [*F*(1, 78) = 0.01, *p* = 0.92; Table 2; Figure 4D]. The main effect of age survived correction for multiple comparisons. Thus, while No-Go RT was faster in older individuals, this pattern did not differ between groups.

### Conflict monitoring was attenuated in some older children with 22q11.2DS

**Figure 5:**
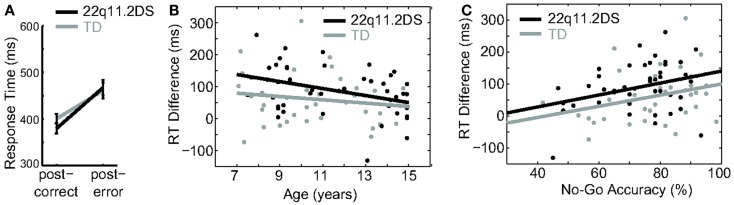
**Conflict monitoring**. **(A)** Both groups demonstrated significant post-error slowing. **(B)** Post-error slowing was negatively associated with age in both groups. **(C)** Post-error slowing corresponded with better reactive inhibition in both groups.

Following detection of a conflict (such as an incorrect response), post-error processing, which is one aspect of conflict monitoring, triggers compensatory adjustments to modulate behavior (31). In speeded response tasks such as this one, it is well established that this manifests as slower RT on the first Go trial following an incorrect relative to a correct No-Go trial. To examine this process in our sample, we regressed diagnostic group, gender, and preceding No-Go trial accuracy on RT for the first Go trial following a No-Go trial. For both groups, RT for Go trials was slower after an incorrect relative to correct No-Go trial, as indicated by a positive difference score [mean RT on the first Go trial following an incorrect minus that following a correct No-Go trial = 60.3(80.0) ms for TD; 87.8(74.4) for 22q11.2DS], and this difference was similar between groups [*F*(1, 80) = 2.58, *p* = 0.11; Figure 5A].

To examine possible age effects on post-error processing, age was regressed on the RT difference score for each group. There was an overall main effect of age, such that age predicted a smaller RT difference score [*F*(1, 78) = 6.59, *p* = 0.02], though this effect did not survive correction for multiple comparisons. Additionally, this relationship did not differ between groups [*F*(1, 78) = 0.69, *p* = 0.41; Table 2; Figure 5B]. To further examine the functional effects of conflict monitoring, age and RT difference score were regressed on No-Go accuracy. There was no Group × Accuracy interaction on difference score [*F*(1, 74) = 0.007, *p* = 0.94; Figure 5C], thus suggesting that better inhibitory performance is related to conflict monitoring similarly in both groups.

### General intellectual ability did not correlate with response inhibition

To examine group-related differences in general intellectual ability, diagnostic group and gender were regressed on FSIQ. Children with 22q11.2DS had significantly lower FSIQ relative to TD children [*F*(1, 55) = 121.7, *p* < 0.0001], an effect that survived after correction for multiple comparisons. In order to examine the possible relationship of this general intellectual ability with response inhibition, we regressed gender and FSIQ on No-Go accuracy for each group. There was no relationship between FSIQ and No-Go accuracy for children with 22q11.2DS [*F*(1, 32) = 1.33, *p* = 0.26] or TD children [*F*(1, 18) = 0.07, *p* = 0.79].

## Discussion

Understanding response inhibition and individual differences in this process in children with 22q11.2DS is of particular importance given their cognitive dysfunction, aberrant neuroanatomy, and high risk for developing schizophrenia in adulthood. We used a Go/No-Go task to examine the nature and development of response inhibition in a cross-sectional study of 7- to 14-year-old children with 22q11.2DS and TD children. Factors that contribute to successful performance on the Go/No-Go task include: (1) *proactive stopping*, which involves appropriate monitoring of the task context and supports preparation leading up to an upcoming inhibitory response; (2) *reactive stopping*, which is the actual implementation of an inhibitory response once instructed by a stop signal; and (3) *conflict monitoring*, which encompasses awareness that an error has been made, and implements appropriate behavioral adjustments in an effort to minimize future errors. We found that children with 22q11.2DS demonstrated proactive stopping comparable to age-matched TD children. By contrast, older children with 22q11.2DS demonstrated aberrant patterns of reactive stopping, and performance varied greatly among these individuals.

The behavioral manifestation of proactive stopping is a relative slowing of responses in preparation for an upcoming inhibitory response. By slowing down, participants are able to stop more successfully if an inhibitory response is required. RT slowing on Go trials did not differ between children with 22q11.2DS and TD children. Thus, children with 22q11.2DS demonstrated typical monitoring of context in anticipation of an increasingly likely No-Go stimulus.

Despite typical proactive stopping, the results suggest aberrant patterns of reactive stopping in children with 22q11.2DS, as well as an impaired developmental trajectory of reactive stopping. While TD children demonstrated better No-Go accuracy on trials that followed a greater number of preceding Go trials, the children with 22q11.2DS did not demonstrate this pattern. Additionally, some older children with 22q11.2DS had worse overall No-Go accuracy relative to older TD children. Interestingly, another study of children in the same age range (and involving a partially overlapping sample of participants from the current study) examined orienting of attention in a Posner cueing paradigm and found the opposite pattern: performance in older individuals with 22q11.2DS was significantly better and less variable than that of their younger counterparts, and no different from that of age-matched TD individuals (35). Therefore, the results of the current study suggest a highly specific age-dependent impairment in reactive inhibitory control, a process that is mediated by neural networks that are known to mature in TD children during this time period (6).

Given the relative specificity of the reactive inhibition impairment in older children with 22q11.2DS, and the relevance of response inhibition in schizophrenia, it is clear that continued exploration of individual performance patterns of response inhibition and their significance in 22q11.2DS is a promising area of further research. After all, these findings generate a number of subsequent questions, such as whether the older, lower-performing individuals are at greater risk for conversion to schizophrenia. If so, performance on this task could be an important contributor as a non-invasive diagnostic measure for risk probability or evaluator of the efficacy of potential targeted interventions (36).

Impairments in response inhibition are common in other neurodevelopmental and neuropsychiatric disorders. In order to examine whether or not group differences in general intellectual ability (non-specific to 22q11.2DS) were driving the results, we included FSIQ as a regressor in the statistical models. We found that FSIQ did not co-vary with task performance in either diagnostic group, suggesting that the results were not driven by IQ differences.

The response inhibition task also enabled us to examine the participants’ abilities to evaluate and modify their own task performance, a process known as conflict monitoring. This evaluative dimension of cognitive control is critical for dispelling the notion of a “homunculus” that guides behavior, as it provides a mechanism for accessing information about how well a system is functioning, enabling behavioral modification based on that information (37). The conflict monitoring hypothesis is supported by evidence that response strategies change reactively during response inhibition tasks, in the form of slower RTs on the first Go trials following incorrect relative to correct No-Go trials (38), an adjustment aimed to generate shifts in speed-accuracy tradeoffs (39). In our study, both children with 22q11.2DS and TD children demonstrated effective post-error slowing, consistent with the conflict monitoring theory. Thus, within the context of this task, it seems that conflict monitoring is relatively unimpaired in children with 22q11.2DS. It will be important to further test this hypothesis through larger studies designed to examine this process specifically.

Importantly, the results of this study generate hypotheses with respect to structural and functional brain abnormalities throughout development in children with 22q11.2DS. Response inhibition is believed to be mediated by circuits involving reciprocal connections between the prefrontal cortex (PFC) and basal ganglia (40, 41). For reactive stopping, sensory input about the stop stimulus projects to the PFC where there are two critical regions for stopping: right inferior frontal cortex (rIFC) and presupplementary motor area (preSMA). These regions then project to the basal ganglia to suppress basal ganglia output that would otherwise initiate motor responses to Go commands (30). Evidence also suggests that this same network is active during proactive stopping (42, 43). For conflict monitoring, evidence supports the role of the anterior cingulate cortex (ACC), which has a modulatory effect on the PFC and is also responsive during the presence of conflict (31).

Though limited, there is some evidence that these circuits are atypical in 22q11.2DS. Structural imaging studies have demonstrated reduction in gray matter of frontal regions in 22q11.2DS (44), as well as alterations in midline cortical thickness and gyrification patterns (45). In one study, reduced volume of ACC was related to poor executive function in children with the disorder (46). There is also evidence for atypical basal ganglia structure in 22q11.2DS (47–49), as well as atypical structural connectivity within frontal networks (50). With respect to more global anatomical differences and their relationship to schizophrenia in 22q11.2DS, one study demonstrated that total brain volume and total white matter volume were reduced in 22q11.2DS adults with schizophrenia, relative to 22q11.2DS adults without schizophrenia (51).

Functional imaging studies have also demonstrated irregularities in these networks in children with 22q11.2DS when compared to TD children. Gothelf et al. (28) examined response inhibition in adolescents with 22q11.2DS using a Go/No-Go task during fMRI, and reported that increased parietal activation might be recruited as a mechanism compensating for atypical prefrontal cortical control of inhibition. Furthermore, atypical prefrontal activity during response inhibition in 22q11.2DS was supported by the finding of reduced No-Go anteriorization (NGA) during a response inhibition task in children and adolescents with 22q11.2DS (52). The NGA is a reliable, stable parameter of medial prefrontal function; thus significantly diminished NGA implies atypical prefrontal control during response inhibition in 22q11.2DS. Interestingly, though the NGA is typically modulated by activation of the ACC, source localization did not identify any alteration of ACC activity during response inhibition in their sample of 22q11.2DS individuals, suggesting that a different mechanism accounted for the observed NGA reduction. Given that the behavioral results of the current study demonstrated that post-error slowing was largely typical in the children with 22q11.2DS, we speculate that conflict monitoring, as mediated by ACC activation, is relatively unimpaired in many of the children with the disorder.

Additional fMRI studies suggest that functional aberrations in neurocognitive networks might contribute to impaired cognitive control and schizophrenia risk in 22q11.2DS. Working memory is another cognitive process that is mediated by the PFC and PFC-associated networks. A study by Kates et al. (53) demonstrated hypoactivation of the PFC during a working memory task in children with 22q11.2DS, in a similar age range as those in our study (8–15 years). More recently, Debbané et al. (54) applied task-independent fMRI to examine high-level neurocognitive networks in adolescents with 22q11.2DS, and found atypical functional connectivity that correlated with prodromal symptoms. Thus, it will be important to pursue neuroimaging studies that directly examine the relationship of neurocognitive network function to response inhibition and schizophrenia risk in 22q11.2DS.

Frontostriatal circuitry follows a protracted developmental time course, proceeding throughout childhood and adolescence, and into early adulthood (55–57), concomitant with the development of response inhibition. Thus, our results suggest the possibility of atypical development of this circuitry in those older individuals with 22q11.2DS who have impaired reactive stopping performance. This interpretation is supported by evidence that the developmental trajectory of cortical gyrification is atypical in children with 22q11.2DS relative to TD children in this age range (6–15 years) (58). The specific nature and timing of these trajectories are still unclear, however, and to date there have only been a few longitudinal studies of developmental trajectories of brain structure in 22q11.2DS (59–62). While these studies indicated neuroanatomical differences in frontal and parietal regions in children and adolescents with 22q11.2DS relative to TD individuals, evidence for atypical developmental trajectories was inconsistent. Larger samples of longitudinal studies during this critical developmental time period will be important for more directly examining the development of brain and behavior relationships responsible for response inhibition in 22q11.2DS.

In addition to brain and behavior relationships, there are a number of additional modulatory factors that are important to consider that will affect individual differences in cognitive control and subsequent risk for psychopathology. These include genetic variants, epigenetic variables, and environmental factors that will all contribute to an individual’s risk for developing schizophrenia, likely as a function of complex interactions of different combinations of the above. One candidate gene of interest within the 22q11.2DS deleted region is the gene for catechol-*O*-methyltransferase (COMT), an important regulator of prefrontal dopamine levels. Since this gene has two possible variants (Val and Met for high versus low enzymatic activity, respectively), some studies have examined the possible relationship of COMT genotype to cognitive function in 22q11.2DS. Results from these studies do not indicate any clear patterns, with some studies reporting Met hemizygosity of COMT to be related to poorer outcome on tasks requiring executive control (63, 64), and others reporting better outcomes (65, 66). Additional studies have found no relationship between COMT genotype and measures of cognitive control in 22q11.2DS (67, 68). The discrepancies are not that surprising, given that the effects of a single gene are not likely to be very powerful, and impact might also vary as a function of other factors such as age, gender (69), or other genetic variants (70).

In summary, a cross-sectional analysis is an important preliminary step for a comprehensive evaluation of the development of response inhibition in children with 22q11.2DS and TD children. When compared to TD children, children with 22q11.2DS do not demonstrate typical developmental improvements in response inhibition. This is suggestive of specific neural and cognitive characteristics that develop differently in children with 22q11.2DS and which may have a critical impact on general neuropsychological impairments and risk for psychopathology in 22q11.2DS.

## Conflict of Interest Statement

The authors declare that the research was conducted in the absence of any commercial or financial relationships that could be construed as a potential conflict of interest.
